# Therapeutic targets for cardiac fibrosis: from old school to next-gen

**DOI:** 10.1172/JCI148554

**Published:** 2022-03-01

**Authors:** Joshua G. Travers, Charles A. Tharp, Marcello Rubino, Timothy A. McKinsey

**Affiliations:** Department of Medicine, Division of Cardiology and Consortium for Fibrosis Research & Translation, University of Colorado Anschutz Medical Campus, Aurora, Colorado, USA.

## Abstract

Cardiovascular diseases remain the leading cause of death worldwide, with pathological fibrotic remodeling mediated by activated cardiac myofibroblasts representing a unifying theme across etiologies. Despite the profound contributions of myocardial fibrosis to cardiac dysfunction and heart failure, there currently exist limited clinical interventions that effectively target the cardiac fibroblast and its role in fibrotic tissue deposition. Exploration of novel strategies designed to mitigate or reverse myofibroblast activation and cardiac fibrosis will likely yield powerful therapeutic approaches for the treatment of multiple diseases of the heart, including heart failure with preserved or reduced ejection fraction, acute coronary syndrome, and cardiovascular disease linked to type 2 diabetes. In this Review, we provide an overview of classical regulators of cardiac fibrosis and highlight emerging, next-generation epigenetic regulatory targets that have the potential to revolutionize treatment of the expanding cardiovascular disease patient population.

## Introduction

Cardiovascular diseases represent the leading cause of death worldwide, accounting for nearly 18 million deaths annually (1). The origins of cardiovascular disease are diverse, predominantly because of the heart’s complexity and the necessity to deliver blood continuously over the course of a lifetime. Cardiac pathology can arise from atherosclerotic vascular disease, obstructive coronary artery disease culminating in myocardial infarction (MI), rhythm abnormalities, valvular dysfunction, cardiac inflammation, primary diseases of the cardiac muscle, and genetic disorders (2). While these etiologies of cardiovascular dysfunction are heterogeneous, a unifying theme in the progression of nearly all forms appears to be the development of cardiac fibrosis. Pathological fibrotic remodeling involves changes in myocardial tissue caused by proliferation and activation of resident cardiac fibroblasts (CFs) and alteration of the extracellular matrix (ECM) composition. While structural collagen is essential for maintaining physiological cardiac function, fibrosis represents pathological changes that correspond with worsened clinical outcomes (3).

The progression of fibrosis from physiological to pathological is perhaps best exemplified by myocardial ischemia and infarction, the most common cause of cardiovascular disease resulting from reduced perfusion of myocardial tissue. Cardiomyocytes, which generate the contractile force mediating cardiac output, require constant energy to maintain function and viability. However, when proper tissue perfusion is lost, cardiomyocytes are deprived of critical sources of energy production, resulting in cell death through either apoptosis or necrosis (4). Myocardial death leads initially to an inflammatory response where granulocytes, macrophages, and fibroblasts are recruited to the region of injury, an area that is ultimately replaced by secreted ECM proteins such as collagen to form scar tissue (5, 6). Reparative scar formation is beneficial in replacing dead cardiomyocytes, preventing myocardial rupture, and maintaining myocardial continuity. However, the replacement of cardiomyocytes with a fibrotic scar following infarction or other forms of cardiac injury reduces contractility and leads to regional or global systolic dysfunction (7). Fibrotic remodeling is also associated with increased passive myocardial stiffness and the development of diastolic dysfunction (DD), an essential contributor to the development of heart failure (HF) with preserved ejection fraction (HFpEF) (8), and can disrupt cardiac electrical conduction by slowing action potential propagation, increasing the risk of arrhythmias and other conduction abnormalities (9).

Despite the substantial contributions of fibrotic remodeling to cardiac dysfunction in both HFpEF and HF with reduced ejection fraction (HFrEF), novel therapies to treat cardiac fibrosis have not emerged in the clinical realm. One principal limitation in the exploration and implementation of antifibrotic therapies stems from the challenge of accurately quantifying fibrotic burden. While definitive diagnosis of cardiac fibrosis by histology is possible, the obtaining of cardiac tissue is limited to invasive endomyocardial biopsy or biopsy during cardiac surgery. Historically, cardiac imaging modalities have not been capable of quantifying cardiac fibrosis. For example, echocardiography, which represents the most common cardiac imaging technique, has not been able to accurately detect fibrosis. In addition, while previous efforts have attempted to correlate diastolic tissue Doppler with collagen deposition by histology, these techniques have not yet been widely adopted (10). Recent advances in magnetic resonance imaging (MRI) technology have allowed for quantification of myocardial ECM volume (11), a technique that has been validated by histological studies of left ventricular (LV) biopsies by Picrosirius red staining (12).

**Figure 1:**
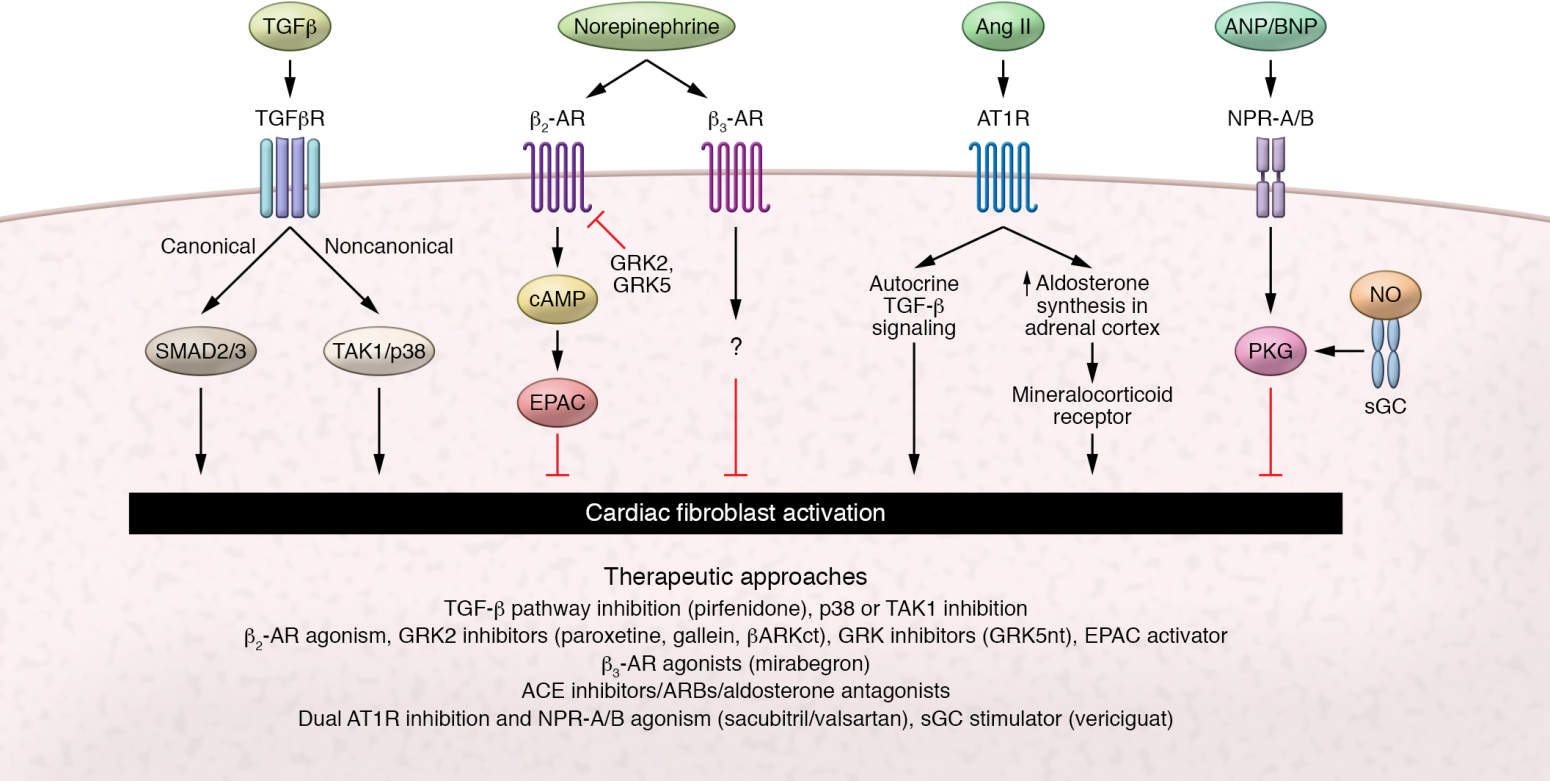
Classical signaling pathways regulating CF activation and approaches for targeting fibrosis of the heart. Numerous signaling pathways have been implicated in the regulation of CF activation and fibrotic remodeling. Therapeutic targeting of these pathways is of intense scientific and clinical interest. TGF-β stimulation of the TGF-β receptor (TGFβR) drives fibroblast activation canonically through SMAD2/3 activation and nuclear translocation, or non-canonically by inducing TGF-β–activated kinase 1–mediated (TAK1-mediated) phosphorylation of p38. While activation of the β_2_-adrenergic receptor (β_2_-AR) is thought to be antifibrotic through induction of cAMP production and activation of exchange protein directly activated by cAMP (EPAC), this signaling can be uncoupled through GPCR kinase 2–mediated (GRK2-mediated) or GRK5-mediated receptor phosphorylation. β_3_-AR agonists, such as mirabegron, may ameliorate fibroblast activation through yet unknown mechanisms. Angiotensin II (Ang II) mediates fibroblast activation through stimulation of the Ang II type 1 receptor (AT1R), by both promoting TGF-β production and inducing systemic release of the mineralocorticoid aldosterone from the adrenal cortex. Induction of cGMP-dependent protein kinase (PKG), through either B-type natriuretic peptide–mediated (BNP-mediated) activation of type A and B natriuretic peptide receptors (NPR-A/B) or stimulation of soluble guanylate cyclase (sGC) by nitric oxide (NO), has also demonstrated antifibrotic properties. Established and investigatory therapeutic strategies targeting these pathways are listed below. ACE, angiotensin-converting enzyme; ARB, angiotensin receptor blocker.

**Figure 2:**
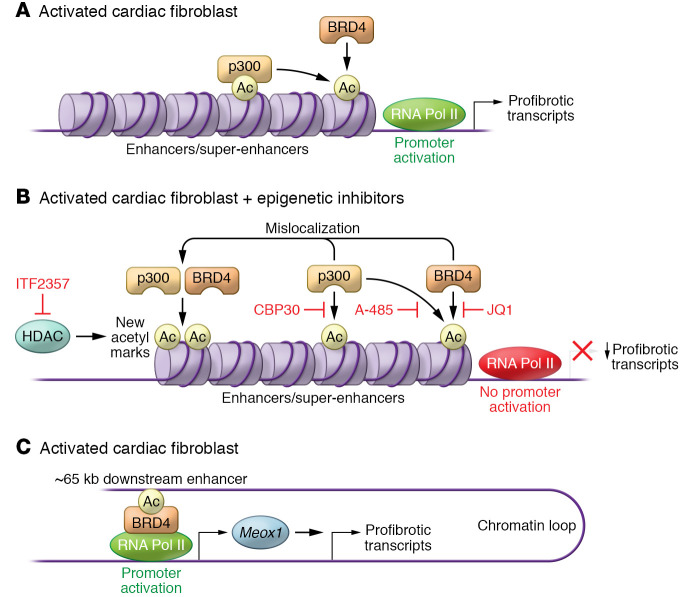
Epigenetic regulation of CF activation and next-generation therapeutic strategies. (**A**) The most notable histone acetyltransferase (HAT) in the control of cardiac fibrosis is p300, which mediates acetylation of histone tail lysine residues in enhancers and super-enhancers that control expression of profibrotic genes. p300 has a bromodomain, which mediates binding of the enzyme to acetyl-histones in chromatin. Bromodomain-containing protein 4 (BRD4) also binds acetyl-histones and initiates a profibrotic gene program by activating RNA polymerase II (RNA Pol II). (**B**) The small-molecule acetyl-lysine mimic JQ1 binds the bromodomains of BRD4 to displace it from chromatin, thereby attenuating profibrotic gene expression. Similarly, CBP30 inhibits the p300 bromodomain, while A-485 inhibits p300 catalytic activity. Pharmacological inhibition of histone deacetylases (HDACs) using compounds such as ITF2357/givinostat creates spurious acetyl-histone marks, resulting in mislocalization of p300 and BRD4 in the cardiac fibroblast genome, with resulting disruption of the profibrotic gene program. (**C**) In activated CFs, BRD4 associates with an enhancer element approximately 65 kb downstream of the gene encoding a homeobox transcription factor, Meox1. Enhancer-bound BRD4 loops to associate with the *Meox1* promoter, resulting in stimulation of its expression and initiation of a profibrotic gene expression cascade.

Understanding, mitigating, and reversing cardiac fibrosis represents a high-yield therapeutic approach for the treatment and prevention of cardiovascular diseases. Here, we provide an overview of classical regulators of cardiac fibrosis (Figure 1), and follow up with an examination of emerging, “next-generation” targets, with an emphasis on epigenetic regulators that we view as having high therapeutic potential (Figure 2). Other exciting advances in this field, such as those related to immune cell– and metabolism-based regulation of cardiac fibrosis, have recently been extensively reviewed elsewhere (13, 14).

## Classical therapeutic targets for cardiac fibrosis

Cardiac fibrosis represents a common terminal pathway seen in diverse cardiac pathologies. The cardiac fibroblast is an essential myocardial cell type responsible for ECM homeostasis; however, stress and pathological stimulation invoke differentiation to a myofibroblast state characterized by increased deposition of ECM proteins, ultimately leading to cardiac fibrosis and dysfunction (3). The cardiac myofibroblast can be activated via numerous cell signaling pathways following cardiac injury, including the transforming growth factor-β (TGF-β) pathway, as well as the adrenergic and angiotensin receptor systems (Figure 1). Below we review these traditional pathways and therapeutic targets.

### TGF-β signaling pathway.

The TGF-β family of peptides represents perhaps the most thoroughly investigated mediator of pathological fibroblast activation and fibrotic remodeling in the heart (15). TGF-β expression is markedly upregulated both in cardiac injury models and in human HF patients (16, 17). Canonical TGF-β signaling involves activin receptor–like kinase 5 (ALK5) and the type II TGF-β receptor (TGFβR2) to mediate activation and nuclear translocation of the profibrotic transcription factors SMAD2 and SMAD3 (18). Recent seminal findings have revealed the critical role for the profibrotic cytokine TGF-β in mediating tissue fibrosis, implicating both TGF-β receptors and SMAD2/3 as principal mediators of resident CF activation and pathological fibrotic remodeling (19). Furthermore, conditional deletion of TGFβR2 in activated myofibroblasts was shown to decrease cardiac fibrosis in mouse models of genetic cardiomyopathy (20, 21), establishing a cell-autonomous role for TGF-β signaling in the control of fibrotic remodeling of the heart. Interestingly, while induction of expression of the secreted matricellular protein connective tissue growth factor (CTGF) has historically been recognized in response to TGF-β stimulation, it now appears as though CTGF is not a major TGF-β effector in the regulation of tissue fibrosis, and unlikely represents a viable therapeutic target (22).

As major players in driving myofibroblast differentiation and pathological ECM deposition, TGF-β and its receptors remain attractive therapeutic targets to combat cardiac fibrosis (23). For example, pharmacological inhibition of ALK5 was recently demonstrated to dedifferentiate cultured cardiac myofibroblasts isolated from failing explanted hearts of transplant recipients, indicating the potential translational feasibility of this approach for ameliorating cardiac fibrosis in human HF patients (24). However, cardiovascular toxicities were recently observed upon general inhibition of the pathway with a TGF-β neutralizing antibody in healthy monkeys (25), suggesting that a more refined approach will be needed to safely inhibit TGF-β signaling to treat human cardiac fibrosis, such as by targeting downstream effectors. In this regard, non-canonical TGF-β signaling, predominantly mediated by TGFβR2, induces activation of TGF-β–activated kinase 1 (TAK1) (26), which represents an attractive therapeutic target as its inhibition has revealed potential antifibrotic effects on TGF-β–induced fibroblast ECM production (27). Further downstream, p38α functions as a nodal regulator of CF differentiation, in part through its ability to drive profibrotic epigenetic regulatory factors to distinct genomic loci (see below). Together, the data suggest that targeted inhibition of p38 may represent a viable therapeutic approach to attenuate myofibroblast activation and fibrosis in response to ischemic injury (28–30).

Pirfenidone is an FDA-approved medication for the treatment of idiopathic pulmonary fibrosis (IPF), which is thought to mediate its effects through a reduction in oxidative stress and TGF-β expression. While the clinical effects of pirfenidone in treating IPF are controversial, a pooled data analysis of randomized clinical trials does reveal clinical benefit (31). In animal models of cardiac fibrosis, pirfenidone was shown to reduce atrial fibrosis, limit fibrotic expansion after infarction, and attenuate hypertension-induced cardiac fibrosis (32–34). In the PIROUETTE clinical trial, in which 47 HFpEF patients were randomized to receive either pirfenidone or placebo, pirfenidone treatment led to a 1.2% decrease in cardiac fibrosis, as determined by MRI assessment of myocardial extracellular volume, but did not improve diastolic function (35); these modest cardioprotective effects are potentially due to redundancy of TGF-β signaling with other pathways that induce pathological fibrotic remodeling and dysfunction, including the adrenergic and angiotensin systems. These data establish a roadmap for using MRI to assess efficacy of antifibrotic therapies for the heart, but also highlight the need to develop more robust interventions to reverse fibrosis and improve clinical outcomes.

### Adrenergic receptor system.

Changes in cardiac physiology in response to stress and reduced cardiac output lead to systemic and local activation of neurohormonal pathways, which are activated to maintain circulatory homeostasis in the setting of cardiac dysfunction and decreased perfusion (36). One important pathway involves activation of the sympathetic nervous system and endogenous release of norepinephrine, a potent, nonselective adrenergic receptor (AR) agonist that stimulates α-ARs in the peripheral vasculature to increase blood pressure, and binds β_1_-ARs in cardiomyocytes to increase inotropy and chronotropy. These effects are beneficial acutely in increasing cardiac output and maintaining adequate blood pressure. However, chronic sympathetic activation becomes maladaptive, and pharmacological blockade of β_1_-ARs remains the most proven therapy for the treatment of HF and prevention of adverse remodeling (37–39).

In contrast to the β_1_-AR, signaling via the β_2_-AR, which appears to be the dominant isoform in CFs, promotes antifibrotic effects, at least in part by activation of exchange protein directly activated by cAMP (EPAC) (40, 41). However, while acute β_2_-AR activation is a potent inhibitor of collagen synthesis in healthy CFs, myofibroblasts isolated from patients with HF appear to be resistant to β_2_-AR agonists, most likely because of receptor uncoupling as a result of elevated GPCR kinase 2 (GRK2) activity (42–44). Targeting GRK2 activity became an attractive antifibrotic strategy following the discovery that its inhibition, pharmacologically with paroxetine (45), or via viral delivery of a peptide inhibitor (βARKct) (46), conferred significant protection against cardiac dysfunction and myocardial fibrosis in numerous animal models of HF. Subsequent studies using genetic ablation of GRK2 specifically in the CF population revealed potent antifibrotic effects in a murine ischemia/reperfusion model of cardiac injury (47, 48), as well as attenuation of myofibroblast differentiation by pharmacological inhibition with gallein (48). The role of GRK5 in cardiac fibrosis has also recently been explored, providing evidence that genetic ablation as well as inhibition using an amino-terminal domain peptide inhibitor (GRK5nt) possesses potential antifibrotic properties (49). Manipulation of GRKs, as they relate to adrenergic signaling in CFs, represents a promising therapeutic strategy to combat myocardial fibrosis.

Unlike β_1_- and β_2_-ARs, the β_3_-AR is thought to be resistant to desensitization because it lacks phosphorylation sites for GRKs (50). β_3_-AR expression is low in nonfailing hearts but is upregulated in response to pathological stress (37, 51–53). Initial evidence of the cardioprotective properties of β_3_-AR signaling was provided by the demonstration that mice lacking this receptor developed exacerbated cardiac remodeling in response to transverse aortic constriction (TAC) due, in part, to augmented nitric oxide synthase–dependent oxidative stress (54). Conversely, β_3_-AR agonists block adverse remodeling in association with reduced cardiac fibrosis in models of pressure overload, MI, and DD (55–57). Based on the beneficial effects of stimulating β_3_-ARs in the heart, mirabegron, a β_3_-AR–selective agonist that is FDA approved for the treatment of overactive bladder, is being assessed for efficacy on LV mass and diastolic function in patients with structural heart disease (58). Future studies to address the mechanisms by which β_3_-AR agonists ameliorate cardiac fibrosis are warranted, including examining whether the antifibrotic effects of β_3_-AR stimulation are due to direct effects on CFs versus indirect effects on cardiomyocytes, endothelial cells, or immune effectors.

### Renin-angiotensin-aldosterone system.

In chronic cardiac dysfunction, there is significant activation of the renin-angiotensin-aldosterone system (RAAS), which has a direct association with the development of cardiac fibrosis (59). As cardiac dysfunction progresses, decreased cardiac output causes a reduction in renal perfusion, stimulating release of renin from the juxtaglomerular apparatus (60). This release leads indirectly to the formation and systemic release of angiotensin II (Ang II) via angiotensin-converting enzyme (ACE). As the integral effector molecule of the RAAS, levels of Ang II are rapidly induced following cardiac injury, and it has been shown to promote numerous myofibroblast characteristics, including elevated cellular proliferation, migration, and ECM synthesis. These effects are secondary to Ang II–mediated stimulation of the Ang II type 1 receptor (AT1R) (61–63), and Ang II’s promotion of TGF-β production in CFs (64).

The benefits of RAAS inhibition in the treatment of HF and prevention of adverse remodeling have been convincingly documented in animal studies as well as human clinical trials. Prevention of Ang II production in patients with HFrEF using ACE inhibitors (ACEIs) reduced hospitalization and all-cause mortality (65). While ACEIs exhibit several salutary effects, including a reduction in blood pressure, there is evidence supporting a direct role in reducing fibrotic tissue burden (66). Similarly, attenuation of Ang II signaling can be achieved using angiotensin receptor blockers (ARBs), which reduce CF activation and block cardiac fibrosis following ischemic insult (67). Interestingly, while global ablation of the Ang II type 1A receptor is cardioprotective following acute MI (68), direct activation of AT1R in cardiomyocytes has only a minimal impact on cardiac hypertrophy (69), suggesting a more important role for this receptor in cardiac nonmyocytes; these data are corroborated by studies demonstrating a reduction in aortic fibrosis in mice lacking AT1R in fibroblasts (70). Clinical trials have also demonstrated improved cardiovascular-related mortality and hospital admissions in patients with HFrEF treated with ARBs (71).

More recently, ARB treatment in combination with a neprilysin inhibitor (NI), which slows degradation of natriuretic peptides, has been shown to function synergistically to reduce fibrosis after experimental ischemic injury (72). Clinically, ARB and NI treatment reduces circulating biomarkers of cardiac fibrosis (73), and was shown to improve outcomes in human patients compared with treatment with ACEIs (74), leading to the FDA approval of sacubitril/valsartan. Multiple lines of evidence have demonstrated that B-type natriuretic peptide (BNP), the levels of which are increased by NI treatment, possesses antifibrotic properties via stimulation of its receptors, NPR-A and NPR-B, with consequent activation of cGMP-dependent protein kinase (PKG) (75). In line with this protective mechanism, vericiguat (76), which elevates cGMP by functioning as a soluble guanylate cyclase (sGC) stimulator, was recently approved for the treatment of HFrEF.

Ang II also promotes cardiac fibrosis indirectly via AT1R activation within the adrenal cortex, inducing systemic release of the mineralocorticoid aldosterone (36). Aldosterone levels are significantly elevated in patients with cardiac dysfunction, leading to increased reabsorption of sodium in the distal convoluted tubule. Aldosterone also possesses direct profibrotic effects in the myocardium, where it induces fibrotic remodeling via activation of mineralocorticoid receptors in CFs (77, 78). Interestingly, the profibrotic effects of aldosterone persist even when the angiotensin system is deactivated, suggesting independent aldosterone-mediated fibrosis (79). Inhibition of aldosterone signaling using the mineralocorticoid receptor antagonists spironolactone and eplerenone has revealed potential antifibrotic effects clinically. The RALES trial, a placebo-controlled study in HFrEF patients, demonstrated a reduction in mortality and hospitalizations following spironolactone administration (80). Furthermore, spironolactone treatment corresponded with reduced serum markers of fibrosis and collagen synthesis (81).

### Progressing beyond the classical regulators of cardiac fibrosis.

Inhibitors of the adrenergic and angiotensin systems are, and will continue to be, a mainstay in the care of the ever-expanding HF patient population. It is important to note, however, that while therapies targeting RAAS appear effective in reducing collagen deposition, recent clinical trials have unfortunately failed to demonstrate benefit in the HFpEF patient population, suggesting an inability to broadly diminish profibrotic pathways (82, 83). In addition, despite evidence supporting the cardioprotective role of pirfenidone, there is an elevated risk of hepatotoxicity associated with prolonged treatment (84), efficacy of the compound in HFpEF patients is modest (35), and the molecular mechanism(s) of action of the compound remain obscure. These disappointing trials may suggest that clinical therapies should be tailored to address the specific etiology underlying cardiac disease, rather than seeking a “universal treatment” for cardiac fibrosis. One possibility for why promising treatments of HFpEF, such as antagonism of angiotensin and aldosterone, have been marginally successful is that beneficial effects may only be observed in a subset a patients in whom fibrosis is driven by the RAAS pathway. In patients who are resistant to such therapy, differential mechanisms regulating CF activation could be operative.

## Targeting epigenetics as an antifibrotic therapeutic approach

Cardiovascular epigenetic mechanisms are rapidly gaining interest for their contributions to the development of myocardial fibrosis and potential to serve as innovative therapeutic targets (85). At its core, epigenetics refers to modifications at the level of chromatin, the basic unit of which is the nucleosome or histone octamer wrapped in DNA, which culminate in alterations in gene expression independent of changes to nucleotide sequence. While the modification varieties are numerous, classical epigenetic events such as acetylation and methylation have been extensively documented for their roles in the pathogenesis of cardiac fibrosis and myofibroblast activation (86). Epigenetic regulators are attractive therapeutic targets since they serve as key nodal points through which redundant upstream pathways, such as those emanating from the cell surface receptors described above, must transmit signals to elicit the gene program for CF activation. Indeed, pharmacological manipulation of several epigenetic modifying enzymes, along with cognate proteins that recognize these modifications and arbitrate differential gene expression, has been shown to mitigate pathological fibrotic remodeling of the heart.

### Histone acetyltransferases.

Acetylation of histone tail lysine residues is a posttranslational modification catalyzed by histone acetyltransferases (HATs). HATs have a profound impact on gene expression, in part by creating docking sites for transcriptional regulators and chromatin-modifying factors that contain acetyl-lysine binding modules, such as bromodomains (87). Among the 28 human HATs (88), the isoform most highly implicated in the control of cardiac fibrosis is p300 (Figure 2A), along with the highly related protein CREB-binding protein (CBP). Nevertheless, efforts to advance HAT inhibitors as a therapeutic strategy for cardiac fibrosis have been hindered by the lack of potent and selective pharmacological inhibitors of p300. The earliest exploration of p300 inhibition in cardiac fibrosis employed curcumin, a natural product HAT inhibitor, which was shown to ameliorate perivascular fibrosis in response to chronic hypertension or following MI (89, 90), as well as reduce ECM production in a high glucose–induced myocardial fibrosis model (91). However, given the pleiotropic actions of curcumin, these data should be approached cautiously. Synthetic p300 inhibitors, such as L002 and C646, have also been shown to block cardiac fibrosis (92–94), but these compounds suffer from a lack of selectivity and potency, respectively.

More recently, through virtual screening and a subsequent medicinal chemistry optimization campaign, A-485, a potent and orally bioavailable small-molecule inhibitor that is highly selective for p300 and CBP, was developed (Figure 2B). A-485 has drug-like properties, providing an excellent opportunity to assess the efficacy of HAT inhibition in preclinical models of pathological cardiac fibrosis, and thereby further address the translational potential of p300/CBP catalytic activity inhibition for the treatment of HF in humans.

p300 and CBP have a single acetyl-lysine binding bromodomain that is required for chromatin targeting of the HATs (95, 96). CBP112 and CBP30 have been developed as small molecules that target the p300/CBP bromodomain and function as acetyl-lysine competitive inhibitors (Figure 2B) (97, 98). Proteomics and transcriptomics were used to quantify acetylation as well as mRNA and protein abundance in mouse embryonic fibroblasts after cellular p300 inhibition with A-485 versus CBP112 (99). Remarkably, gene expression changes triggered by CBP112 were modest compared with those observed upon catalytic inhibition of p300 with A-485, suggesting that the HAT bromodomain is required for the regulation of only a subset of target genes. To our knowledge, p300/CBP bromodomain inhibitors have yet to be tested in models of cardiac fibrosis. However, the recent demonstration that CBP30 potently blocks activation of fibroblasts from patients with Dupuytren’s disease, a localized fibrotic disorder of the palm, suggests antifibrotic potential of this approach (100).

### Histone deacetylases.

Seemingly paradoxically, inhibiting deacetylation of histones by targeting histone deacetylases (HDACs) also blocks cardiac fibrosis. Mammalian HDACs are divided into four classes: class I (HDAC1, HDAC2, HDAC3, and HDAC8), class II (HDAC4, HDAC5, HDAC6, HDAC7, HDAC9, and HDAC10), class III (SIRT1–7), and class IV (HDAC11). Class II is further subdivided into IIa (HDAC4, HDAC5, HDAC7, and HDAC9) and IIb (HDAC6 and HDAC10). Class I, II, and IV HDACs are zinc-dependent, while class III HDACs (also known as sirtuins) use NAD^+^ as a cofactor for catalytic activity. We focus on the potential of inhibiting zinc-dependent HDACs for the treatment of cardiac fibrosis. Nonetheless, it should be noted that sirtuins clearly regulate fibrosis of the heart, but that their activity is generally cardioprotective (101, 102), and thus therapeutic strategies should likely focus on stimulating sirtuin activity. Consistent with this, enhancing sirtuin catalytic activity by providing animals with NAD precursors or by stimulating NAD biosynthesis was shown to improve diastolic function in murine models of HFpEF (103, 104).

The ability of “pan” inhibitors of zinc-dependent HDACs, such as the FDA-approved compound suberoylanilide hydroxamic acid (SAHA; vorinostat), to block cardiac fibrosis in MI, TAC, and genetic models of systolic HF has been well documented and reviewed extensively (105, 106). More recently, it was shown that pan-HDAC inhibition with the clinical-stage compound ITF2357/givinostat improved cardiac relaxation in murine models of hypertension- or aging-induced DD with preserved ejection fraction, and that SAHA was efficacious in a feline model of HFpEF due to slow, progressive ascending aortic banding (107–109). Surprisingly, in the murine models of DD, cardiac fibrosis was not observed by standard histological readouts, such as Picrosirius red staining (107, 108), and improved diastolic function upon HDAC inhibition was attributed exclusively to augmented myofibril relaxation (107). However, further evaluation of hearts of mice with DD revealed “hidden fibrosis,” a process in which increased ECM deposition and remodeling were not detected by standard histological methods, but were uncovered by quantitative mass spectrometry and atomic force microscopy (AFM). This covert type of cardiac fibrosis was profoundly inhibited by ITF2357/givinostat in a manner that correlated with improved diastolic function (108), implicating HDAC inhibition as a potential therapeutic strategy to combat HFpEF induced by pathological ECM remodeling and resulting ventricular stiffening.

Employing histological methods to assess the role of fibrosis in the pathogenesis of human HFpEF has yielded equivocal findings. In one autopsy study, individuals with HFpEF were shown to have more pronounced cardiac fibrosis than control subjects (110), while in an independent study using endomyocardial biopsies from HFpEF patients with severe DD, approximately 30% of the samples examined did not have significant fibrosis (111). Reevaluation of these human and murine hearts, as well as additional samples from HFpEF patients and preclinical models, using ECM mass spectrometry and AFM should more clearly define whether ECM expansion serves a generalizable role in the control of DD and HFpEF.

There are two pressing questions related to HDAC inhibitor–mediated inhibition of cardiac fibrosis: (a) Which HDAC isoforms are profibrotic? (b) What are the molecular mechanisms by which these enzymes promote fibrosis? Regarding the first question, hydroxamic acid pan-HDAC inhibitors such as SAHA and givinostat are far more effective at blocking the catalytic activity of class I and IIb HDACs than class IIa HDACs (112), which have catalytic domains but no known physiological substrates (113), or class IV HDAC11, which is a lysine defatty acylase as opposed to a deacetylase (114–116). While nothing is known about the cardiac function of HDAC10 (117), which is a spermidine deacetylase, knockout of HDAC6 had no effect on cardiac fibrosis (118), suggesting that class IIb HDACs are not generally profibrotic. Thus, class I HDACs are likely the targets of SAHA and givinostat that promote cardiac fibrosis. Consistent with this, selective class I HDAC (HDAC1, -2, -3) inhibition with mocetinostat blocked cardiac fibrosis in response to chronic Ang II infusion in mice (119), and blunted progression of fibrosis in a chronic rat MI model, resulting in a reduction in LV end-diastolic pressure (120). Furthermore, in a 7-day model of mouse MI, induced by left anterior descending coronary artery ligation, administration of the class I HDAC inhibitor PD-106 after MI resulted in reduced LV remodeling and improved cardiac function at study endpoint, with concomitant suppression of matrix metalloproteinase-2 and -9 expression (121).

It remains possible that other HDAC isoforms serve profibrotic roles in the heart that have gone unnoticed owing to reagent limitations. In this regard, new, highly selective inhibitors of class IIa HDACs or HDAC11 catalytic domains have been developed, and should be employed to assess the roles of these obscure HDACs in the control of cardiac fibrosis (122, 123).

Surprisingly little is known about the molecular mechanisms by which HDAC inhibitors block cardiac fibrosis. Class I HDAC inhibition has been shown to suppress CF proliferation by preventing retinoblastoma protein (Rb) phosphorylation, thereby preventing expansion of ECM-producing myofibroblasts (119). Class I HDAC inhibition was also shown to stimulate expression of antifibrotic microRNA-133 (miR-133), leading to suppression of TAC-mediated cardiac fibrosis in mice (124). More recently, suppression of CF activation by HDAC inhibition was linked to mislocalization of the chromatin “reader” protein bromodomain-containing protein 4 (BRD4) (see below) (108).

### Bromodomain and extraterminal proteins.

The bromodomain and extraterminal (BET) family of proteins, BRD2, BRD3, BRD4, and BRDT, associate with acetylated lysine residues of histones to regulate gene transcription. BRD4 and BRDT (testis-specific) possess carboxy-terminal domains capable of activating RNA polymerase II (Pol II) through the positive transcription elongation factor (P-TEFb) complex to initiate gene transcription (125, 126). While several small-molecule inhibitors of BET proteins have been developed, the best characterized is JQ1, an acetyl-lysine mimetic that competitively displaces BET bromodomains from chromatin, resulting in suppression of Pol II–mediated transcription (Figure 2B) (127). JQ1 prevented several hallmarks of HF, including cardiomyocyte hypertrophy, cardiac fibrosis, and systolic dysfunction, in a mouse model of TAC (128, 129), and in a model of genetic dilated cardiomyopathy caused by a mutant form of phospholamban (PLNR9C) (130). Furthermore, administration of JQ1 in a therapeutic mode after the heart had remodeled also attenuated cardiac dysfunction both in the murine TAC model and in post-MI cardiac remodeling in mice (131).

Integrated transcriptomic analyses across rodent HF models and human induced pluripotent stem cell systems have clearly revealed that BET inhibition suppresses transactivation of a broad profibrotic and proinflammatory gene program in the heart (131). Mechanistically, BRD4 is known to contribute to the formation of dynamic, cell state–specific enhancers, referred to as super-enhancers (SEs). BRD4 disproportionately associates with acetyl-H3K27–containing SEs, which are thought to signal proximal promoters to stabilize BRD4-containing coactivator complexes near transcription start sites, and thereby facilitate P-TEFb–mediated Pol II phosphorylation and transcription elongation (132–134). In CFs, TGF-β signaling targets BRD4 binding to discrete SEs in a p38 kinase–dependent manner, providing a circuit for coupling extracellular cues to the cardiac epigenome to drive profibrotic gene expression (135). Subsequent studies, using single-cell technologies, identified distal regulatory elements in CFs that had increased chromatin accessibility after TAC that were closed upon JQ1 treatment (136). One of the most highly regulated elements was a large enhancer downstream of the gene encoding Meox1, a homeodomain-containing transcription factor whose expression was highly upregulated in myofibroblasts after TAC and suppressed by JQ1. Regulation of Meox1 expression in CFs involved TAC-inducible association of the *Meox1* promoter with BRD4 bound to this enhancer region, which is located approximately 65 kb downstream (Figure 2C). Follow-on studies with cultured CFs established a new role for Meox1 as a profibrotic transcription factor in the heart. Thus, these studies uncovered a stress-inducible, BRD4-dependent, long-range chromatin interaction as an important, druggable regulator of cardiac fibrosis.

A recent study determined chromatin quantitative trait loci in human hearts by assessing H3K27 acetylation by ChIP-Seq and follow-up chromatin conformation assays (137). The work identified 62 putative enhancers with increased H3K27 acetylation enrichment, corresponding gene expression differences, and overlap with published subthreshold GWAS hits, suggesting potential disease and phenotype association. Given the propensity of BRD4 to associate with acetyl-H3K27, it is intriguing to speculate that BET inhibitors could target these loci to block HF pathogenesis.

BRD4 may also regulate cardiac fibrosis by mediating crosstalk between myocytes and fibroblasts or other nonmyocyte populations in the heart. ChIP-Seq studies revealed that, in addition to controlling pro-growth genes, many of the BRD4-enriched SEs identified in cardiomyocytes were associated with profibrotic genes, including those encoding the secreted factors CTGF, plasminogen activator inhibitor-1 (PAI-1/Serpine1), and TGF-β2 (138). These findings suggest the possibility that BRD4 signaling in cardiomyocytes regulates expression of paracrine factors that activate fibroblasts and other stress-activated cell types in the heart to elicit fibrotic remodeling.

BET proteins contain tandem bromodomains, BD1 and BD2, which are simultaneously targeted by inhibitors such as JQ1. Emerging evidence exploring other inhibitors, such as the BD2-selective inhibitor apabetalone, suggests that inhibiting one BRD4 bromodomain over the other may improve the overall safety profile for HF patients requiring chronic therapy. Indeed, apabetalone is the only BET inhibitor to be tested in a phase III trial for any indication, being assessed for its ability to reduce major cardiovascular events in more than 2400 individuals with combined acute coronary syndrome (ACS), type 2 diabetes (T2D), and low LDL levels. While apabetalone failed to diminish ischemic cardiovascular events in this patient population, the BD2-selective inhibitor was found to be well tolerated, and secondary subgroup analyses revealed a reduction in hospitalizations for HF in patients with T2D and recent ACS (139), and fewer HF-related hospitalizations in patients with chronic kidney disease and T2D (140). Thus, the feasibility of safely targeting BRD4 as a therapeutic strategy for cardiovascular disease is established.

How is it that inhibition of HATs, HDACs, or BET proteins results in inhibition of CF activation? Clearly there is crosstalk between these epigenetic regulatory factors (Figure 2B). HAT activity is required to create acetyl-marks at profibrotic enhancers that are subsequently bound by BRD4. Furthermore, there is evidence demonstrating that HDAC inhibition, which creates spurious acetyl-histone marks, results in mislocalization of BRD4 in the CF genome (108) and prevents HATs from properly acetylating certain gene regulatory elements in the heart (141), which may also involve altering genomic targeting of bromodomain-containing HATs.

### Only the tip of the epigenetics iceberg.

We have focused much of this Review on a single epigenetic modification, acetylation, and a small number of regulators of the posttranslational modification. However, it is important to note that other mediators of the epigenome have been shown to regulate fibrosis of the heart. For example, genetic ablation or pharmacological inhibition of the K3K9me2-specific demethylase KDM3a was shown to diminish collagen deposition in the mouse TAC model (142), and myofibroblast-specific ablation of lysine-specific demethylase 1 (LSD1/KDM1) was found to alleviate systolic dysfunction and fibrosis in the TAC model by broadly interdicting pathological TGF-β1 signaling (143). Furthermore, the vast majority of epigenetic regulatory factors have yet to be studied in the context of cardiac fibrosis and HF, underscoring a deep reservoir for basic and translational research discoveries that have the potential to profoundly impact patients suffering from various cardiovascular diseases.

**Figure 3:**
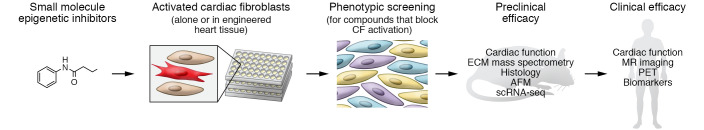
Discovering the next generation of antifibrotic epigenetic inhibitors for the heart. Proposed model for expeditiously uncovering novel epigenetics-based therapeutics targeting fibroblast activation and cardiac fibrosis. The recent development of highly selective and potent inhibitors of myriad epigenetic targets has laid a strong foundation for therapeutic investigation using ex vivo, imaging-based phenotypic screening and subsequent exploration in in vivo models of cardiac fibrosis and HF. Given the existence of “hidden fibrosis,” histological assessment of fibrotic remodeling of the heart should be complemented with techniques such as ECM mass spectrometry, atomic force microscopy (AFM), and single-cell RNA sequencing. We envision that these approaches will allow desperately needed therapeutic strategies targeting myofibroblast activation and fibrotic remodeling to finally bridge the gap to the clinical realm.

It is our view that the most expeditious path forward is to blend genetic and pharmacological, “chemical biology” approaches. In this regard, exhaustive and sophisticated medicinal chemistry programs in industry and academia have led to the development of highly selective and potent inhibitors of a wide array of epigenetic targets, and many of these compounds are available to the scientific community through programs such as the Structural Genomics Consortium (144). Coupling the use of these compounds with well-validated ex vivo phenotypic assays and in vivo models of cardiac fibrosis has the potential to rapidly uncover novel roles for epigenetic regulators in the control of HF, providing crucial mechanistic insights, and to advance lead compounds into in vivo efficacy go/no-go experiments in the march toward the clinic (Figure 3). However, we acknowledge that this stance could be viewed as “old school,” since we have not touched on other exciting therapeutic modalities, such as gene editing, RNA, or antibody therapies, or the promising discovery that chimeric antigen receptor T cells engineered to specifically target activated fibroblasts are able to reduce cardiac fibrosis in a mouse model (145).

Finally, identification of the optimal therapeutic window for targeting cardiac fibrosis will be paramount for effective treatment. First, premature disruption of reparative scar formation holds significant risk for cardiac rupture, as is observed when physiological fibroblast function is disturbed too abruptly following infarction (146–148). Similarly, a key therapeutic concern for antifibrotic therapies relates to ECM maturity, specifically the point in a disease process at which the matrix has become so heavily cross-linked that it is potentially no longer degradable. At least in regard to cell therapy, there exists a “point of no return” following ischemic injury when the infarct scar has reached a mature state and the cardioprotective effects may no longer be possible (149). In this regard, in addition to targeting fibroblast activation and ECM deposition, approaches aimed at enhancing turnover of the fibrotic matrix in the heart should also be pursued.

## Conclusions

While standard-of-care medications have proven invaluable in the fight against cardiovascular diseases over the last several decades, there remains a critical need to pursue novel therapeutic strategies targeting fibroblasts and fibrotic remodeling. A considerable amount of research effort is now dedicated to exploring myriad exciting and promising lines of investigation to combat cardiac fibrosis, including expansion of classical regulators of fibrosis as well as more novel strategies in the area of epigenetics. Furthermore, incredible technological advancements in our ability to probe complex cellular systems and screen compound libraries for antifibrotic agents will undoubtedly prove instrumental in driving this field forward. Innovative therapeutic interventions targeting cardiac myofibroblasts and the pathological fibrotic remodeling they promote have high potential to lead to advancements in the treatment of human cardiovascular diseases.

## Version 1 03/01/2022

electronic publication
